# Oleanolic Acid Ameliorates Hepatic Lipid Metabolism and Autophagy in Type 2 Diabetic Mice via the STAT3 Signaling Pathway

**DOI:** 10.1002/fsn3.72063

**Published:** 2026-07-15

**Authors:** Zirui Ji, Yuanfeng Huang, Xiangjin Pu, Jinqing Feng, Yiwen Liao, Wanfen Lin, Qing Zhu, Weixuan Wang

**Affiliations:** ^1^ Traditional Chinese Medicine Research Institute Guangdong Pharmaceutical University Guangzhou Guangdong China; ^2^ Guangdong Provincial Research Center of Integration of Traditional Chinese Medicine and Western Medicine in Metabolic Diseases Guangzhou Guangdong China; ^3^ Key Laboratory of Glucolipid Metabolic Diseases Ministry of Education Guangzhou Guangdong China; ^4^ Guangdong Provincial TCM Key Laboratory for Metabolic Diseases Guangdong Pharmaceutical University Guangzhou Guangdong China

**Keywords:** autophagy, lipid metabolism, oleanolic acid, STAT3, type 2 diabetic mellitus

## Abstract

Type 2 diabetes mellitus (T2DM) poses a major global health challenge due to its considerable disability and mortality rates. The complex etiology of T2DM involves hepatic steatosis and autophagy dysfunction. Previous research indicates that oleanolic acid (OA) ameliorates T2DM, with signal transducer and activator of transcription 3 (STAT3) serving as a crucial regulator in numerous cellular processes. Therefore, this study aims to explore whether the therapeutic effects of OA are associated with STAT3 activation and its potential role in hepatic lipid metabolism and autophagy in T2DM. T2DM was induced in mice by feeding a high‐fat diet followed by multiple low‐dose streptozotocin injections. OA notably lowered blood glucose and lipid levels, along with improvements in glucose tolerance and insulin sensitivity. Additionally, OA alleviated liver injury and steatosis. In T2DM mice, OA reduced hepatic protein and gene expression associated with fatty acid uptake/synthesis while enhancing those linked to fatty acid β‐oxidation. Moreover, OA modulated autophagy‐related proteins, as evidenced by increased p62 levels and decreased LC3‐II and Beclin‐1 levels. Molecular docking analysis predicted a potential interaction between OA and STAT3, and Western blot showed increased STAT3 phosphorylation at Y705 after OA treatment*.* In vitro, STAT3 inhibition blunted the ability of OA to restore the expression profiles of autophagy and lipid metabolism in insulin‐resistant liver cells. Collectively, OA may ameliorate T2DM via concomitant STAT3 phosphorylation to modulate hepatic lipid metabolism and autophagy. This study offers novel insights into the mechanisms by which OA treats T2DM, presenting fresh perspectives for T2DM clinical management.

## Graphical abstract

This study demonstrates that oleanolic acid (OA) may ameliorate type 2 diabetes mellitus (T2DM) by modulating hepatic lipid metabolism and autophagy in a manner concomitant with STAT3 phosphorylation. Specifically, OA downregulates fatty acid synthesis/uptake‐related proteins (SREBP‐1, ACC, CD36), upregulates fatty acid β‐oxidation‐related protein (PPARα), and modulates autophagy‐related proteins (p62, LC3‐II, Beclin‐1), all of which are associated with STAT3 phosphorylation.

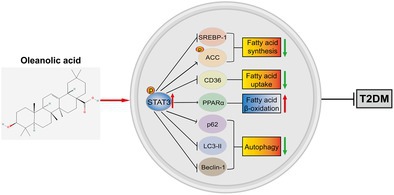

## 1 Introduction

Diabetes mellitus, a chronic metabolic disorder, severely diminishes the quality of life for patients, escalates mortality rates, and increases healthcare costs, posing a significant threat to global health. By 2050, the prevalence of diabetes is projected to rise to 12.95%, affecting approximately 853 million individuals (Federation 2025). Type 2 diabetes mellitus (T2DM), accounting for over 90% of diabetes cases, is estimated by the International Diabetes Federation to be associated with 3.4 million deaths in 2024, making it a major contributor to global mortality (Federation 2025). Timely pharmacological intervention is necessary for managing poorly controlled blood glucose levels in the progressive disease of T2DM (Jia et al. 2019). Therefore, investigating the pathogenesis of T2DM and developing effective drugs to alleviate the disease is essential to meet a critical clinical need.

As a chronic metabolic disorder, T2DM is hallmarked by hyperglycemia, insulin resistance, and impaired insulin secretion (Sadeghi et al. 2023). Despite its intricate etiology not being completely understood, current evidence underscores that disturbances in autophagy and lipid metabolism play a pivotal role in driving T2DM progression (Sadeghi et al. 2023). Among these, ectopic fat deposition within the liver is recognized as a critical contributor. (Sadeghi et al. 2023). Hepatic lipid metabolism is a finely regulated process. Hepatic steatosis arises when the intake of fatty acids and the process of *de novo* lipogenesis (DNL) surpass the rate of their secretion and oxidation. Liver steatosis development is marked by a notable rise in hepatic uptake of fatty acids and DNL. Initially, fatty acid oxidation is compensatorily enhanced, yet it remains insufficient to significantly decrease lipid buildup. As hepatocellular metabolic functions progressively deteriorate, the liver's lipid export capacity may stabilize or even decrease, leading to continuous lipid accumulation and triggering systemic metabolic disorders (Ipsen et al. 2018). Excessive fatty acid uptake leads to lipid metabolism imbalance, causing hepatic steatosis and the accumulation of toxic lipids (Nie et al. 2023). Fatty acid accumulation in the liver triggers oxidative stress, increases chronic inflammatory responses, and ultimately induces hepatic insulin resistance (Ipsen et al. 2018). Insulin resistance further exacerbates lipid metabolism disorders, creating a vicious cycle. In insulin‐resistant states, hepatic DNL rises, fatty acid oxidation diminishes, and the influx of fatty acids from adipocytes grows, resulting in triglyceride accumulation in the liver (Moon 2017). These studies highlight the importance of exploring lipid metabolism disorders in T2DM, highlighting potential directions for future research.

Autophagy represents a critical intracellular degradative mechanism through which cytoplasmic constituents‐including proteins and organelles‐are delivered to lysosomes via autophagosomes. Upon fusion with lysosomes, autophagosomes become autolysosomes, wherein the sequestered cargo is subsequently degraded (Chao et al. 2020). A substantial body of evidence has identified autophagy as a key regulator of systemic homeostasis, particularly in the context of hepatic physiology and disease (Mizushima and Komatsu 2011; Sadeghi et al. 2023). Dysregulated autophagy is linked to metabolic disorders including T2DM, renal disease, and cardiomyopathy (Sadeghi et al. 2023). As a central mediator of longevity‐associated signaling pathways, autophagy is recognized for its role in enhancing life span through the promotion of cellular regeneration and by mitigating inflammation and oncogenic risks (Mizushima and Komatsu 2011). However, autophagy is a double‐edged sword. Besides its cytoprotective functions, it can also exhibit cytotoxic, non‐protective, and cytostatic effects (Gewirtz 2014). In pancreatic β‐cells, enhanced autophagosome formation has been noted in both C57BL/6 mice on a high‐fat diet (HFD) as well as *db/db* mice, indicating that high caloric intake or obesity‐related insulin resistance may trigger β‐cell autophagy (Ebato et al. 2008). The accumulation of autophagic vacuoles and autophagosomes in the β‐cells of patients with T2DM may lead to reduced β‐cell mass (Masini et al. 2009), whereas diminished autophagy lowers proinsulin degradation and boosts insulin secretion (Riahi et al. 2016). Therefore, autophagy is closely associated with the progression of T2DM, yet the precise mechanisms warrant further investigation.

Signal transducer and activator of transcription 3 (STAT3), a cytoplasmic transcription factor, is phosphorylated at tyrosine 705 (Y705), triggering its homodimerization, movement into the nucleus, and binding to DNA. This process stimulates the transcription of downstream target genes involved in cell growth, differentiation, blood vessel formation, inflammation, etc. (H. Lee et al. 2019). Studies have demonstrated a tight association between STAT3 and metabolic diseases, including T2DM and non‐alcoholic steatohepatitis (NASH). STAT3 is involved in various metabolic diseases, including T2DM and non‐alcoholic steatohepatitis (NASH). In mice with liver‐specific STAT3 knockout, hepatic gluconeogenic gene expression is elevated, accompanied by insulin resistance. In contrast, overexpression of STAT3 in diabetic mice reduces gluconeogenic gene expression and markedly lowers both blood glucose and plasma insulin levels (Inoue et al. 2004). Additionally, enhanced STAT3 phosphorylation has been shown to mitigate hepatic lipid metabolic disturbances, alleviate inflammation, and improve insulin sensitivity in mice with HFD‐induced NASH (Cai et al. 2016). Our earlier research showed that STAT3 activation improves glucose uptake, promotes glycolysis, and suppresses gluconeogenesis, effectively ameliorating abnormal liver glucose metabolism in T2DM mice (Wang, Liang, et al. 2023). Therefore, STAT3 is crucial in T2DM progression, necessitating further investigation into its mechanisms.

STAT3 is crucial in regulating lipid metabolism and autophagy. Research indicates that dimethyl fumarate inhibits adipogenic differentiation by suppressing STAT3 activity in 3 T3‐L1 preadipocytes, leading to reduced expression of lipogenic transcription factors and cell cycle proteins (Kang et al. 2013). In *Il‐10* gene‐knockout mice fed with ethanol or a HFD, the elevation of IL‐6 activates STAT3, thereby alleviating alcoholic and non‐alcoholic liver steatosis and hepatic injury (Miller et al. 2011). By preventing STAT3 activation, mice with adipocyte‐specific JAK2 deletion impede lipolysis and promote weight gain, resulting in age‐related insulin resistance (Shi et al. 2014). Additionally, studies have shown that STAT3 has a negative regulatory effect on autophagy, especially in the occurrence of tumors (Xu et al. 2022). In U937 lymphoma cells under starvation, IL‐6 addition significantly increases STAT3 phosphorylation at Y705 and decreases autophagy levels, whereas the STAT3 inhibitor LLL12 boosts levels of autophagy‐associated proteins LC3 and Beclin‐1 (Qin et al. 2013). The novel microtubule‐disrupting compound CYT997 is capable of triggering autophagy by silencing the JAK2/STAT3 pathway in gastric cancer cells (Cao et al. 2020). These studies indicate that during the progression of T2DM, the STAT3 signaling could be pivotal in regulating lipid metabolism and autophagy.

Oleanolic acid (OA) represents one of the most widely distributed pentacyclic triterpenoids in the human diet, occurring in more than 1600 plant species, with olives and olive oil constituting its most important dietary sources—providing an approximately 25 mg daily intake in the Mediterranean diet. Additionally, OA is abundant in the peel of various common fruits including jujube, apple, and grape, as well as in the fruit of *Ligustrum lucidum* , a traditional Chinese medicine. Such widespread dietary and medicinal exposure positions OA as a critical candidate linking routine consumption to metabolic health (Günther and Bednarczyk‐Cwynar 2025; Žiberna et al. 2017). It exhibits a range of biological activities, including hepatoprotection, antioxidant properties, anti‐inflammatory effects, and hypoglycemic properties (Wang and Liu 2024). These characteristics endow OA with great potential for application in the medical field, attracting extensive attention from researchers. In particular, its potential in treating diabetes mellitus and related complications is supported by numerous studies. Research has shown that OA ameliorates insulin resistance in the liver of *db/db* mice by exerting anti‐inflammatory effects, reducing lipid levels, and inhibiting mitochondrial ROS (Wang et al. 2013). OA may lower blood lipid and glucose concentrations in rats with alloxan‐induced diabetes, possibly due to its antioxidant properties (Gao et al. 2009). Moreover, OA can enhance insulin sensitivity, metabolic function, and reduce hepatic steatosis in obese rats via the gut‐liver axis, employing anti‐inflammatory, antioxidant, and probiotic mechanisms (Xue et al. 2021). Accordingly, previous studies point out the multidimensional effects of OA in treating T2DM, establishing a basis for its therapeutic potential. Further research is still needed to elucidate the underlying mechanisms and identify key targets of OA to facilitate the development of targeted treatment strategies for T2DM.

This study shows that OA can improve T2DM by regulating liver lipid metabolism in T2DM mice and insulin‐resistant HepG_2_ cells. And interestingly, we elucidated the regulatory effects of OA on autophagy, which has been rarely studied. Furthermore, we are the first to show that OA's effects may be mediated through the STAT3 pathway. By identifying the association between STAT3 phosphorylation and OA's effects, our study illuminates the mechanisms of OA in treating T2DM, offering new perspectives for the clinical prevention and treatment of T2DM, as well as for the development of new pharmaceuticals.

## 2 Materials and Methods

### 2.1 Resources

The HFD was obtained from Dyets (HF60, Wuxi, China). Streptozotocin (STZ, S0130) was sourced from Sigma‐Aldrich (St. Louis, MO, USA). Metformin hydrochloride (Met, H20023371) was obtained from Sino‐American Shanghai Squibb Pharmaceuticals Ltd. (Shanghai, China). Sodium carboxymethyl cellulose (CMC‐Na, 30036365) was supplied by Sinopharm Chemical Reagent Co. Ltd. (Shanghai, China). Stattic (S7024) was procured through Selleck Chemicals (Houston, TX, USA). Oleanolic acid was sourced from Chengdu LeMeiTian Pharmaceutical Technology Co. Ltd. (Chengdu, China; HPLC ≥ 98%). Phosphatase inhibitor (P1081), protease inhibitor (P1045), and glucosamine (S1635) were acquired from Beyotime (Shanghai, China). Insulin (PB180432) and glucose were respectively obtained from Procell Life Science & Technology Co. Ltd. (Wuhan, China) and Fresenius Kabi SSPC (Wuxi, China). The CCK‐8 assay kit (MA0218‐2) was procured from meilunbio (Dalian, China).

Elabscience (Wuhan, China) provided kits for measuring high‐density lipoprotein cholesterol (HDL‐C) (E‐BC‐K221‐M) and low‐density lipoprotein cholesterol (LDL‐C) (E‐BC‐K205‐M). Meanwhile, the Nanjing Jiancheng Bioengineering Institute (Nanjing, China) supplied kits for measuring aspartate aminotransferase (AST, C010‐2‐1), alanine aminotransferase (ALT, C009‐2‐1), total cholesterol (TC, A111‐1‐1), triglyceride (TG, A110‐1‐1), and glucose (A154‐1‐1). Anti‐β‐Actin antibody (EM21002) was sourced from HUABIO (Hangzhou, China). Anti‐STAT3 (60199‐1‐Ig) antibody was obtained from Proteintech (Chicago, IL, USA). Antibodies including anti‐p‐STAT3 (9145S), anti‐p‐ACC (11818), anti‐ACC (3676), and anti‐CD36 (74002S) were obtained from Cell Signaling Technology (Danvers, MA, USA). Anti‐CD36 antibody (bs‐8873R) was purchased from Bioss (Beijing, China). Anti‐p62 antibody (AF5384) was purchased from Affinity Biosciences (China). Anti‐LC3 antibody (T55992M) and anti‐Beclin‐1 antibody (T55092) were procured from Abmart (Shanghai, China). The anti‐SREBP‐1c antibody (Sc‐365513) was sourced from Santa Cruz (Dallas, TX, USA). Anti‐PPARα antibody (Ab178865) was sourced from Abcam (Cambridge, UK).

### 2.2 The Design of Animal Studies

C57BL/6 mice (male, 6 weeks old) were sourced from Zhuhai BesTest (Zhuhai, China). Mice were kept in an SPF barrier system with temperature regulation at 25°C ± 2°C, with a simulated natural photoperiod, and where access to food and water was either unrestricted or appropriately restricted according to experimental requirements. The animal testing protocols received approval from the Guangdong Pharmaceutical University's Animal Ethics Committee (Approval Number gdpulacspf2022195).

**FIGURE 1:**
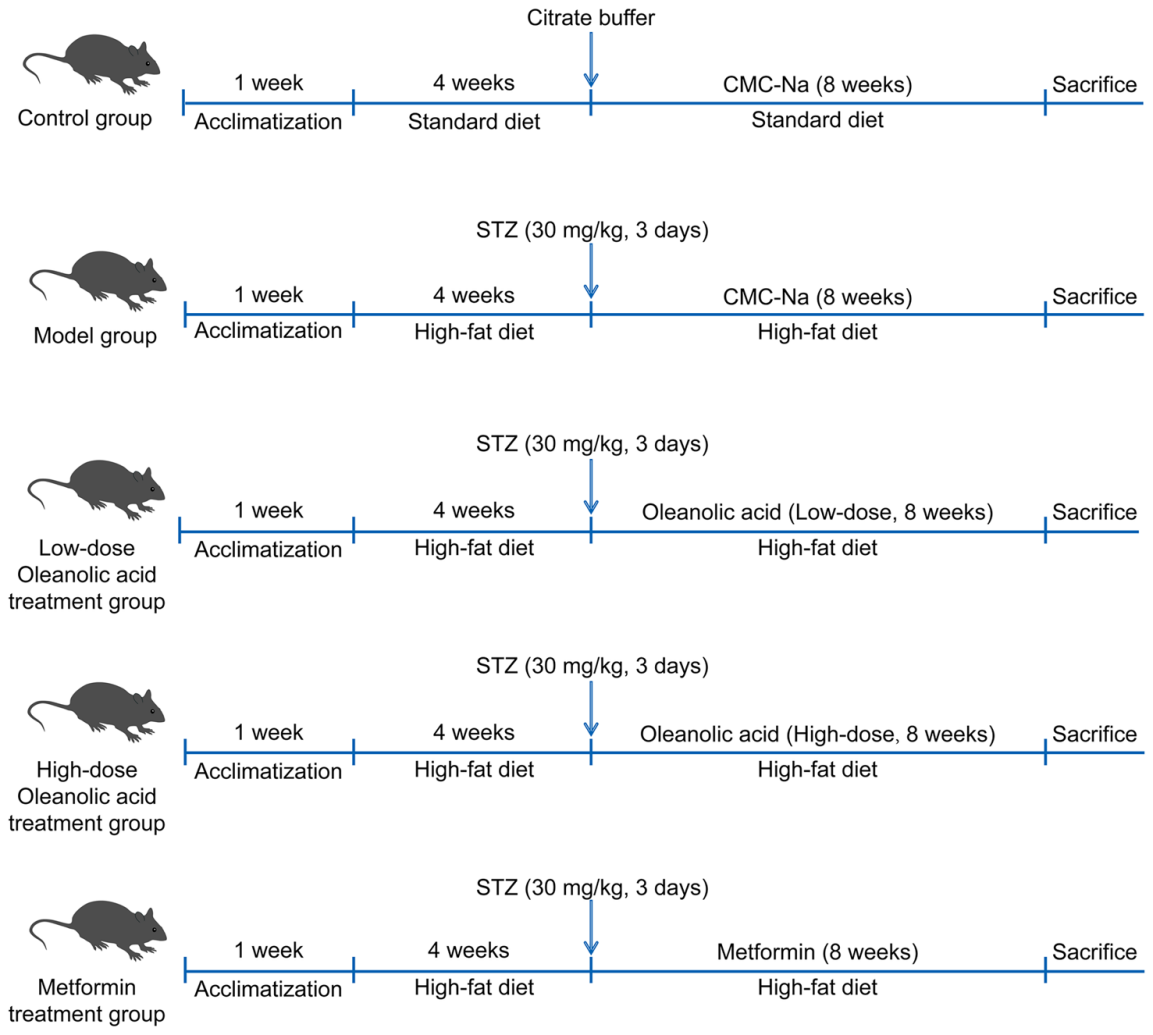
Animal experimental flowchart.

The control group (Ctrl, *n* = 8) was fed a standard diet. After a one‐week adaptive feeding period, a T2DM murine model was established by administering a HFD followed by streptozotocin (STZ) injection (Wang, Liang, et al. 2023). STZ was dissolved in 0.1 M citrate buffer to prepare a stock solution at pH 4.2–4.5. The final injection dose was adjusted to 30 mg/kg body weight based on individual mouse weight. After 4 weeks on a HFD, mice received daily intraperitoneal injections of this stock solution for three consecutive days, while continuing the HFD. Mice with fasting blood glucose levels over 11.1 mmol/L were classified as having T2DM (Dai et al. 2020; Wang, Liang, et al. 2023). The successfully established T2DM mice were subsequently allocated into 4 groups using a random number table: the model group (Mod, *n* = 8), the low‐dose OA‐treated group (OA‐L, *n* = 8, 20 mg/kg/day), the high‐dose OA‐treated group (OA‐H, *n* = 8, 60 mg/kg/day), and the metformin‐treated group (Met, *n* = 8, 250 mg/kg/day) (Chen et al. 2015; Dai et al. 2020; Wang et al. 2019; Wang, Liang, et al. 2023). Compared with non‐treatment groups receiving vehicle alone, mice in the treatment groups received daily gavage with OA or metformin throughout the 8‐week period. Finally, after a 12 h fast, blood was harvested from the mice under isoflurane anesthesia via the retro‐orbital venous plexus. Deep isoflurane anesthesia was then maintained until humane euthanasia was completed by cervical dislocation. Refer to Figure 1 for the animal experimental flowchart.

### 2.3 The Oral Glucose Tolerance Test (OGTT) and the Intraperitoneal Insulin Tolerance Test (IPITT)

**FIGURE 2:**
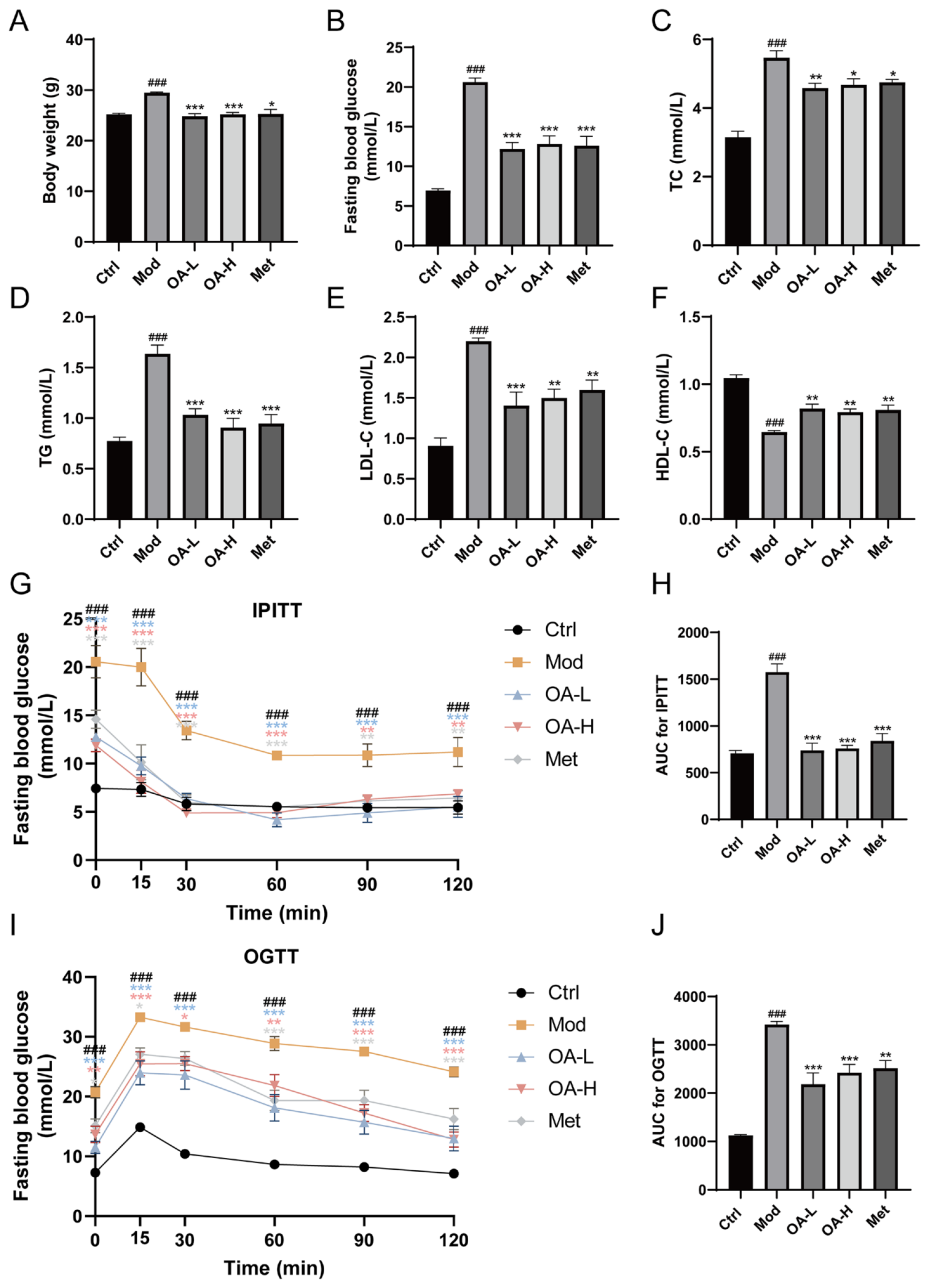
Oleanolic acid (OA) improves glucose and lipid metabolism in Type 2 Diabetes Mellitus (T2DM) mice. (A) Body weight, (B) Fasting blood glucose, (C) Serum total cholesterol (TC), (D) Serum triglycerides (TG), (E) Serum low‐density lipoprotein cholesterol (LDL‐C), and (F) Serum high‐density lipoprotein cholesterol (HDL‐C) levels. (G) The intraperitoneal insulin tolerance test (IPITT). (H) Area under the curve (AUC) for the IPITT. (I) The oral glucose tolerance test (OGTT). (J) AUC for the OGTT. *n* = 6, values were expressed as mean ± SEM. ^###^ *p <* 0.001 vs. the Ctrl group; *p < 0.05, **p < 0.01, ***p < 0.001 vs. the Mod group.

OGTT and IPITT were performed 1 week apart during the treatment period, prior to euthanasia. For both tests, mice were fasted for 6 h prior to the procedures. Oral glucose (2 g/kg) was administered via gavage, whereas insulin (0.75 U/kg) was given by intraperitoneal injection. Blood glucose levels were determined at the time points indicated in Figure 2G,I using a Johnson & Johnson glucometer (Milpitas, CA, USA). All blood samples were drawn from the tail vein to ensure efficient and accurate data collection.

### 2.4 Biochemical Evaluation of Serum and Liver Specimens

Blood samples were stood at room temperature for a full hour. Following centrifugation at 3500 rpm under low‐temperature conditions lasting 15 min, the resulting supernatant, which served as the test sample, was aliquoted and stored in an ultra‐low temperature freezer at −80°C.

Liver specimens (50 mg) were homogenized with 450 μL of physiological saline. After centrifugation, the homogenates were thoroughly mixed, and a BCA Protein Assay Kit (Beyotime Biotechnology, Shanghai, China) was employed for protein concentration determination.

Measurements were performed according to the manufacturer's protocols for serum parameters (Figure 1C–F), liver function markers (Figure 2A,B), and hepatic lipid contents (Figure 2D,E).

### 2.5 Histopathological Staining

Section the paraffin‐embedded liver tissue into 4 μm thin sections and mount them on slides. After deparaffinization, the cell nuclei in the sections were colored by hematoxylin. Then, sections were briefly rinsed to remove excess stain. Counterstaining was performed using eosin, after which the specimens were dehydrated by sequential immersion in graded ethanol solutions (80%, 95%, 100%, and 100%). Prior to mounting, the sections were transferred to xylene for transparency. Microscopic examination (Olympus, Tokyo, Japan) was utilized to observe cellular structures and pathological changes.

Section frozen liver tissue into 10 μm thin sections and mount them on slides. After fixation, sections underwent a brief wash, followed by staining with Oil Red O under light‐protected conditions. Quickly rinse off excess stain with 60% isopropanol, followed by counterstaining with hematoxylin for 10 min. Finally, coverslip with glycerin gelatin. The stained sections were examined microscopically to evaluate lipid deposition (Olympus). To minimize observer bias, sections were coded with random numbers prior to examination by an investigator blinded to group allocation. The severity of hepatic steatosis was qualitatively assessed across multiple fields of view, and representative images were subsequently selected to illustrate typical lipid accumulation.

### 2.6 Molecular Docking

The three‐dimensional structure of OA was retrieved from the PubChem database (CID: 10494). The crystal structure of STAT3 (PDB ID: 6NJS) was downloaded from the RCSB Protein Data Bank. This structure was selected based on the following quality criteria: resolution < 3.0 Å and R‐value free < 0.3, and it meets these standards with a resolution of 2.70 Å and an *R*‐value free of 0.256. Protein preparation, including the removal of water molecules and the addition of polar hydrogens, was performed using AutoDock Tools (version 4.2). Both the receptor and ligand were subsequently converted into PDBQT format.

Based on literature research, three potential druggable pockets within the SH2 domain of STAT3 were identified: the pY +0 pocket (key residues: Arg609, Ser613, Lys591), the pY +1 pocket (key residues: Phe588, Ile589, Ser590, Val637), and the hydrophobic side pocket (key residues: Phe716, Met660, Pro715, Ser636) (Kong et al. 2021). The grid box centers (*x*, *y*, *z*) and dimensions were set as follows: for the pY +0 pocket (Pocket 1) – (*x*: 8.4, *y*: 44.7, *z*: −2.2; size: 23.8 × 17.8 × 24.8 Å); for the pY +1 pocket (Pocket 2) – (*x*: 3.7, *y*: 47.2, *z*: 0.9; size: 23.5 × 26.9 × 23.2 Å); and for the hydrophobic side pocket (Pocket 3) – (*x*: 9.8, *y*: 60.0, *z*: −2.9; size: 18.0 × 31.5 × 13.4 Å).

To validate the docking protocol, the co‐crystallized ligand SD36 was extracted from the crystal structure and re‐docked into its original binding site using AutoDock Vina with the same parameters as described above (exhaustiveness = 100, num_modes = 24, energy_range = 3 kcal/mol). The grid box was centered at the coordinates of the co‐crystallized ligand (*x*: 13.5, *y*: 54.1, *z*: 0.1) with dimensions of 12.0 × 21.7 × 14.0 Å. Clustering analysis of the redocking poses within an RMSD cutoff of 2.0 Å showed that the conformational cluster representing the crystal binding mode was successfully enriched at a favorable energy level (−8.1 kcal/mol). The best near‐crystal pose achieved an RMSD of 1.88 Å, validating the conformational sampling reliability of the docking protocol under extensive search space.

### 2.7 Cell Culture and Treatment

HepG2 human liver cells were sourced from the Shanghai National Collection of Authenticated Cell Cultures (Shanghai, China) and maintained in DMEM with 10% fetal bovine serum (FBS; Gibco, Waltham, MA, USA) and 1% penicillin/streptomycin (Wisent, Montreal, QC, Canada). It was through exposure to 18 mM glucosamine for 18 h that HepG2 cells were rendered insulin‐resistant (Wang, Liang, et al. 2023). Following this, cells were incubated for 24 h with OA (75 μM), with or without Stattic (1.5 μM), under glucosamine conditions (Jafari et al. 2020).

### 2.8 Western Blot Analysis

Liver samples and HepG2 cells underwent lysis in RIPA buffer (Solarbio), with a blend of 1% protease inhibitors and 1% phosphatase inhibitors. After quantification and denaturation, protein samples were resolved by SDS‐PAGE. Subsequently, the proteins were transferred to a PVDF membrane using the wet transfer method, followed by blocking with 5% skim milk. Primary antibodies, except for Beclin‐1 and LC3, which were diluted 1:500, were diluted 1:1000. After overnight incubation with the membranes at 4°C, the membranes were exposed to secondary antibodies diluted to 1:2000 for 1 h at room temperature, followed by five additional TBST washes. The membranes were treated with ECL chemiluminescent reagent (Yeasen, Shanghai, China). A gel imaging system (Bio‐Rad, Hercules, CA, USA) was applied for visualizing protein bands, while the ImageLab software (Bio‐Rad) was utilized for the quantitative analysis of protein expression level.

### 2.9 Real‐Time Quantitative Polymerase Chain Reaction (qPCR)

RNA extraction from liver tissues and HepG2 cells, reverse transcription, and qPCR were performed using commercial kits (TRIzol, PrimeScript RT, and SYBR Green; Takara, Japan) as previously reported (Wang, Liang, et al. 2023). The qPCR analysis was performed using a LightCycler 480 (Roche, Basel, Switzerland), with β‐Actin as the normalization control. The 2^−ΔΔCT^ method was employed for data analysis. Primer sequences, designed and synthesized by Ruibiotech (Beijing, China), are detailed in Table S1 (Supporting Information S1).

### 2.10 Statistical Analysis

Data analysis was performed using GraphPad Prism 8 software, with results presented as mean ± SEM. Sample sizes were determined based on technical quality control considerations and standard laboratory practices: physiological and biochemical analyses were standardized to *n* = 6 per group after excluding hemolyzed endpoint serum samples (which interfere with colorimetric assays) and OGTT/IPITT data compromised by severe stress responses (e.g., during repeated handling, oral gavage, intraperitoneal injections, and consecutive tail vein blood collections, resulting in instantaneous blood glucose fluctuations). Hepatic gene expression was analyzed with *n* = 8 per group as liver tissue was unaffected by serum hemolysis. Western blot and histological examinations were conducted with three representative samples per group following standard laboratory practice. The normality of data distribution was assessed using the Shapiro–Wilk test, and homogeneity of variances was evaluated using both Bartlett's test and Brown‐Forsythe test. For datasets that satisfied both normality and homogeneity of variances, parametric tests were applied: comparisons between two groups were performed using an unpaired two‐tailed Student's *t*‐test, and comparisons among multiple groups were performed using one‐way or two‐way analysis of variance (ANOVA) followed by Tukey's post hoc test for multiple comparisons. For datasets that met the normality assumption but exhibited heterogeneity of variances, the Brown‐Forsythe ANOVA test followed by Dunnett's T3 post hoc test was used. For datasets that did not meet the normality assumption, the non‐parametric Kruskal‐Wallis test followed by Dunn's post hoc test was applied. A *p* < 0.05 signified statistically significant differences between groups.

## 3 Results

### 3.1 OA Improves Glucose and Lipid Metabolism in T2DM Mice

To evaluate the therapeutic potential of OA in T2DM, a mouse model was induced by administering a HFD combined with STZ, followed by 8 weeks of treatment with OA or the positive control metformin. Compared with the Ctrl group, the T2DM mice exhibited significantly higher body weight and fasting blood glucose levels (Figure 2A,B), confirming the successful establishment of the diabetic model. Administration of OA or metformin led to a significant reduction in these measures (Figure 2A,B). Furthermore, serum levels of TC, TG, and LDL‐C in T2DM mice were elevated, yet they were notably reduced following treatment with OA or metformin, while the reduced levels of HDL‐C were raised with the same treatments (Figure 2C–F). OGTT and IPITT results demonstrated that both OA and metformin treatments enhanced insulin sensitivity and glucose tolerance (Figure 2G–J). Furthermore, OA treatment significantly mitigated HFD/STZ‐induced abnormal pancreatic enlargement (Figure S1). Collectively, these data suggest that OA can ameliorate the dysregulated glucolipid metabolism in T2DM mice.

### 3.2 Treatment With OA in T2DM Mice Ameliorates Liver Damage and Steatosis

**FIGURE 3:**
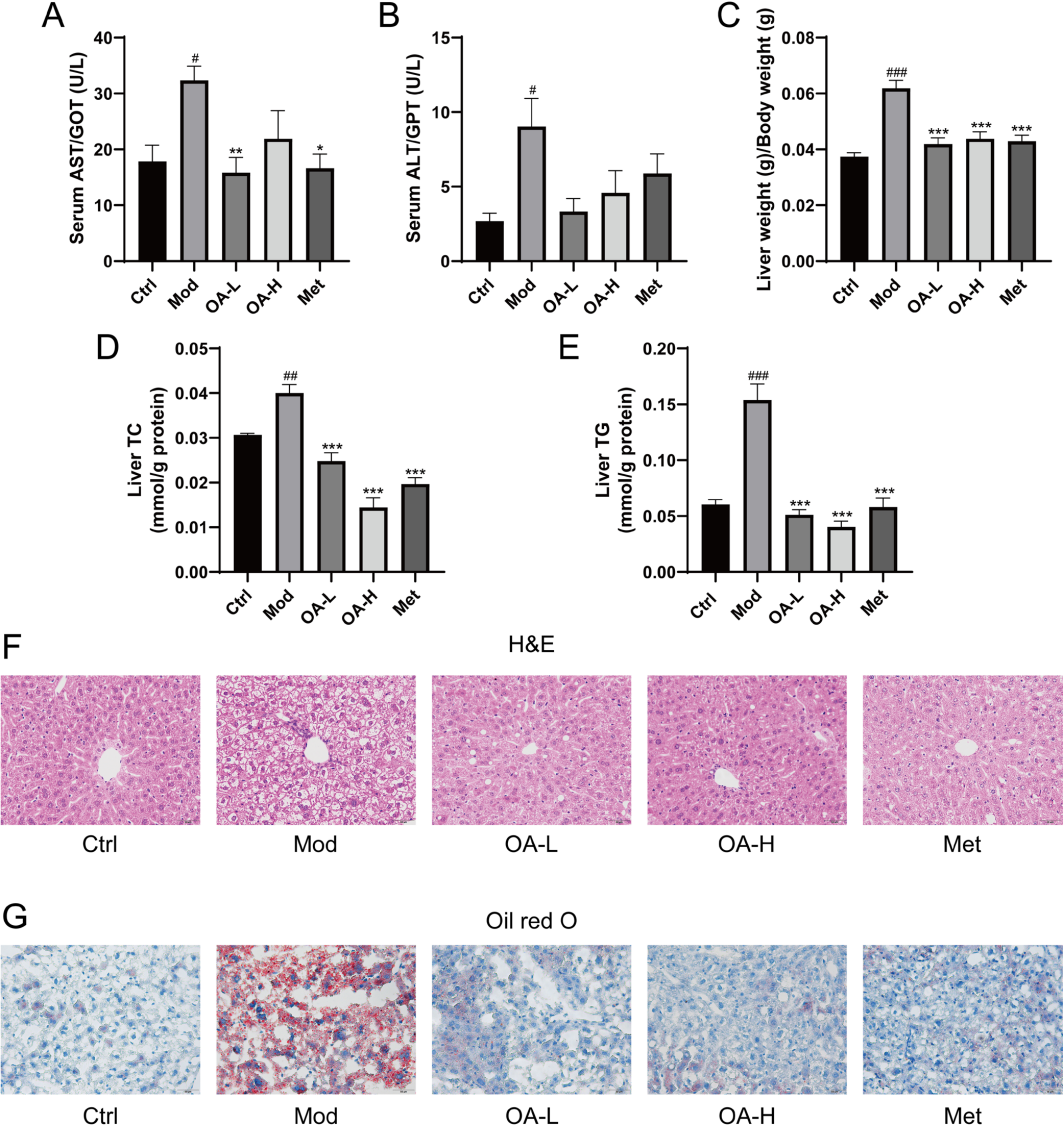
Treatment with OA in T2DM mice ameliorates liver damage and steatosis. The activities of (A) serum aspartate aminotransferase (AST) and (B) alanine aminotransferase (ALT). (C) Liver weight/Body weight. The levels of liver (D) TC and (E) TG. *n* = 6 in (A–E). (F, G) Representative histological images of mouse liver sections stained with hematoxylin–eosin (H & E) and Oil Red O. Scale bar = 50 μm. Data are expressed as mean ± SEM. #*p* < 0.05, ^##^ *p* < 0.01, ^###^ *p* < 0.001 versus the Ctrl group; *p < 0.05, **p < 0.01, ***p < 0.001 versus the Mod group.

The liver is of great importance for regulating fat metabolism and maintaining lipid homeostasis in the T2DM condition (Ipsen et al. 2018). Therefore, this study will concentrate on the liver to enhance the pathophysiological understanding of T2DM. In T2DM group, there was a marked elevation in hepatic injury indicators, namely ALT and AST, surpassing those observed in the Ctrl group (Figure 3A,B). Furthermore, versus controls, the T2DM group demonstrated a pronounced rise in liver index along with hepatic TC and TG levels (Figure 3C–E). Subsequent intervention with OA or Met elicited distinct ameliorative effects. None of the three regimens conferred a statistically significant improvement in serum ALT levels, despite apparent reductions in the OA‐treated groups. OA‐L significantly suppressed the AST surge, whereas OA‐H merely exhibited a downward trend without attaining statistical significance. Both OA regimens significantly diminished the elevated liver index. Additionally, OA‐H manifested superior potency in reducing hepatic TC and TG content relative to OA‐L. Met comparably attenuated AST aberration and lipid accumulation; however, its impact on serum ALT remained equally non‐significant (Figure 3A–E). Then, H & E staining indicated that Mod group mice contained numerous unevenly distributed fat vacuoles, disrupted hepatic cell cord arrangement, compromised cellular morphological structure integrity, and inflammatory cell infiltration (Figure 3F). Oil Red O staining indicated a profusion of lipid droplets within the hepatic tissues of Mod mice (Figure 3G). Treatment with OA or metformin significantly reversed the aforementioned conditions (Figure 3F,G). These data indicate that OA significantly ameliorates liver damage and steatosis in T2DM mice.

### 3.3 OA Reduces Fatty Acid Uptake and Synthesis While Enhancing β‐Oxidation in the Livers of T2DM Mice

**FIGURE 4:**
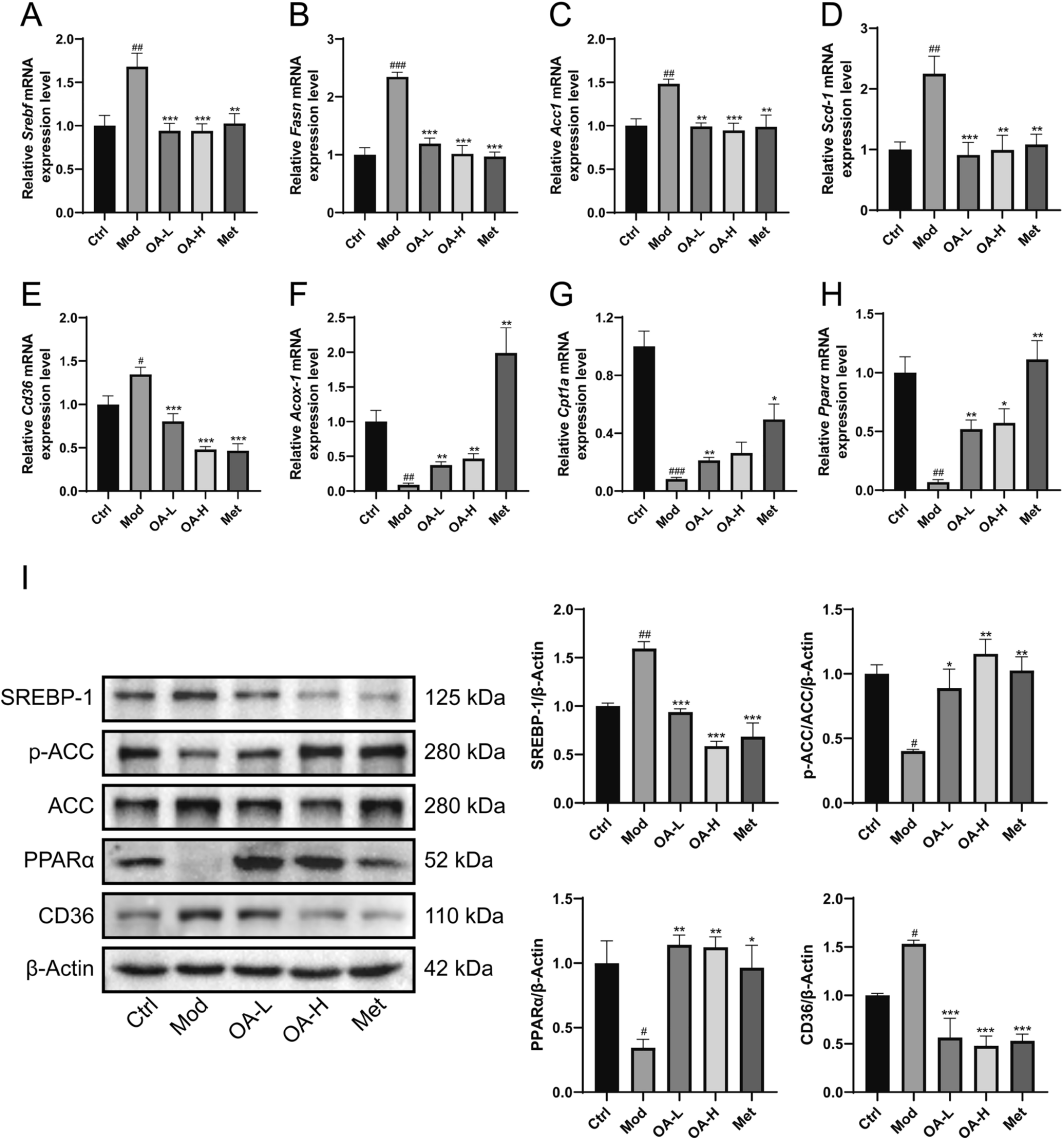
OA reduces hepatic fatty acid uptake and synthesis while enhancing β‐oxidation in T2DM mice. (A–D) The hepatic expression of genes involved in fatty acid synthesis. (E) The hepatic expression of *Cd36*. (F–H) The hepatic expression of genes linked to fatty acid β‐oxidation. *n* = 8 in (A–H). (I) The hepatic protein levels of SREBP‐1, p‐ACC, ACC, PPARα, CD36, and β‐Actin were evaluated, with a bar graph presenting the quantitative western blot data. β‐Actin served as a loading control, *n* = 3. Data were expressed as mean ± SEM. ^#^ *p* < 0.05, ^##^ *p* < 0.01, ^###^ *p* < 0.001 versus the Ctrl group; *p < 0.05, **p < 0.01, ***p < 0.001 versus the Mod group.

Alterations in the expression of lipid‐related genes were analyzed to understand how OA counteracts hepatic steatosis. Relative to the Ctrl group, the Mod group exhibited significant upregulation of genes associated with fatty acid synthesis, namely *Srebf*, *Fasn*, *Acc1*, and *Scd‐1*. (Figure 4A–D). In the Mod group, *Cd36* expression, which is linked to fatty acid uptake, increased relative to the Ctrl group (Figure 4E), whereas genes including *Acox‐1*, *Cpt1a*, and *Pparα*, which are linked to fatty acid β‐oxidation, were notably down‐regulated (Figure 4F–H). Treatment with OA or metformin reversed the above‐mentioned changes (Figure 4A–H). Western blot analysis corroborated the qPCR results, showing that the Mod group exhibited a marked decrease in ACC phosphorylation at Ser79 indicating elevated ACC activity, markedly augmented protein expression of SREBP‐1 and CD36, and a notable reduction of PPARα protein levels in contrast to the Ctrl group (Figure 4I). Following OA and metformin treatment, SREBP‐1 and CD36 levels were notably decreased, meanwhile ACC phosphorylation and PPARα protein levels were significantly up‐regulated (Figure 4I). These results indicate that OA may ameliorate dysregulated hepatic lipid metabolism in T2DM mice, likely through modulation of multiple fatty acid metabolism‐related pathways.

### 3.4 OA Alters Autophagy‐Related Protein Expression in the Livers of T2DM Mice

**FIGURE 5:**
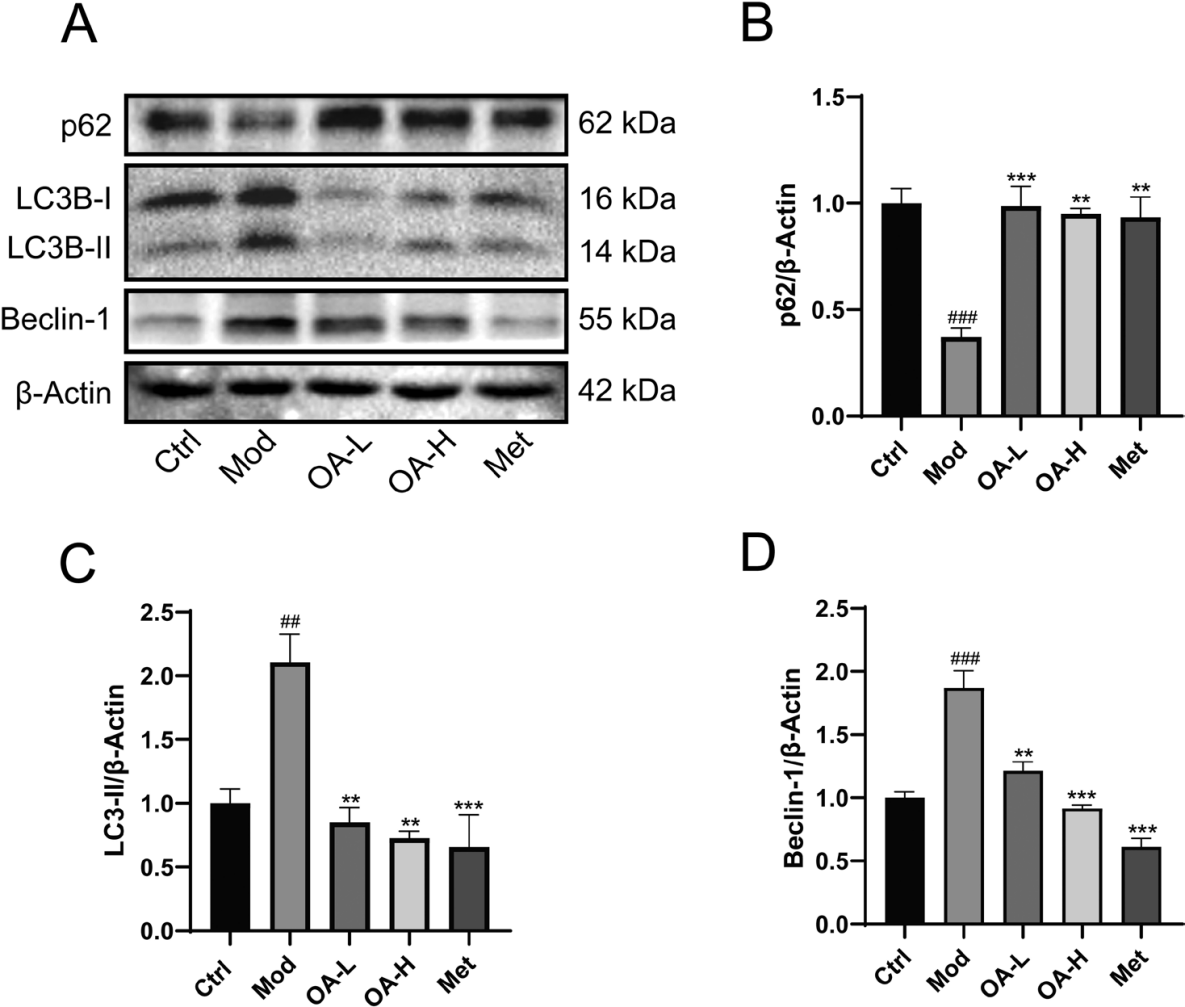
OA alters autophagy‐related protein expression in the livers of T2DM mice. Western blot was conducted to quantify the hepatic expressions of p62, LC3, Beclin‐1, and β‐Actin. β‐Actin served as a loading control. (B–D) Bar graphs illustrating quantification results obtained from western blot experiments. *n* = 3. Data are expressed as mean ± SEM. ^##^ *p* < 0.01, ^###^ *p* < 0.001 versus the Ctrl group; **p < 0.01, ***p < 0.001 versus the Mod group.

Abnormal autophagy has a correlation with multiple metabolic disorders, such as T2DM (Sadeghi et al. 2023). Western blot analysis of autophagy‐linked proteins revealed that the Mod group displayed substantially diminished hepatic levels of the autophagy substrate p62 and up‐regulated levels of Beclin‐1 and LC3‐II compared to the Ctrl group (Figure 5A–D). Following treatment with OA and metformin, p62 expression significantly increased, whereas Beclin‐1 and LC3‐II expressions markedly decreased (Figure 5A–D). The above‐mentioned data show altered expression of autophagy‐related proteins in T2DM mice livers, with OA treatment partially reversing these changes. However, without direct measurement of autophagic flux, it remains unclear whether these changes reflect enhanced autophagy initiation or impaired lysosomal degradation.

### 3.5 Improvement of T2DM by OA Is Associated With STAT3 Phosphorylation

**FIGURE 6:**
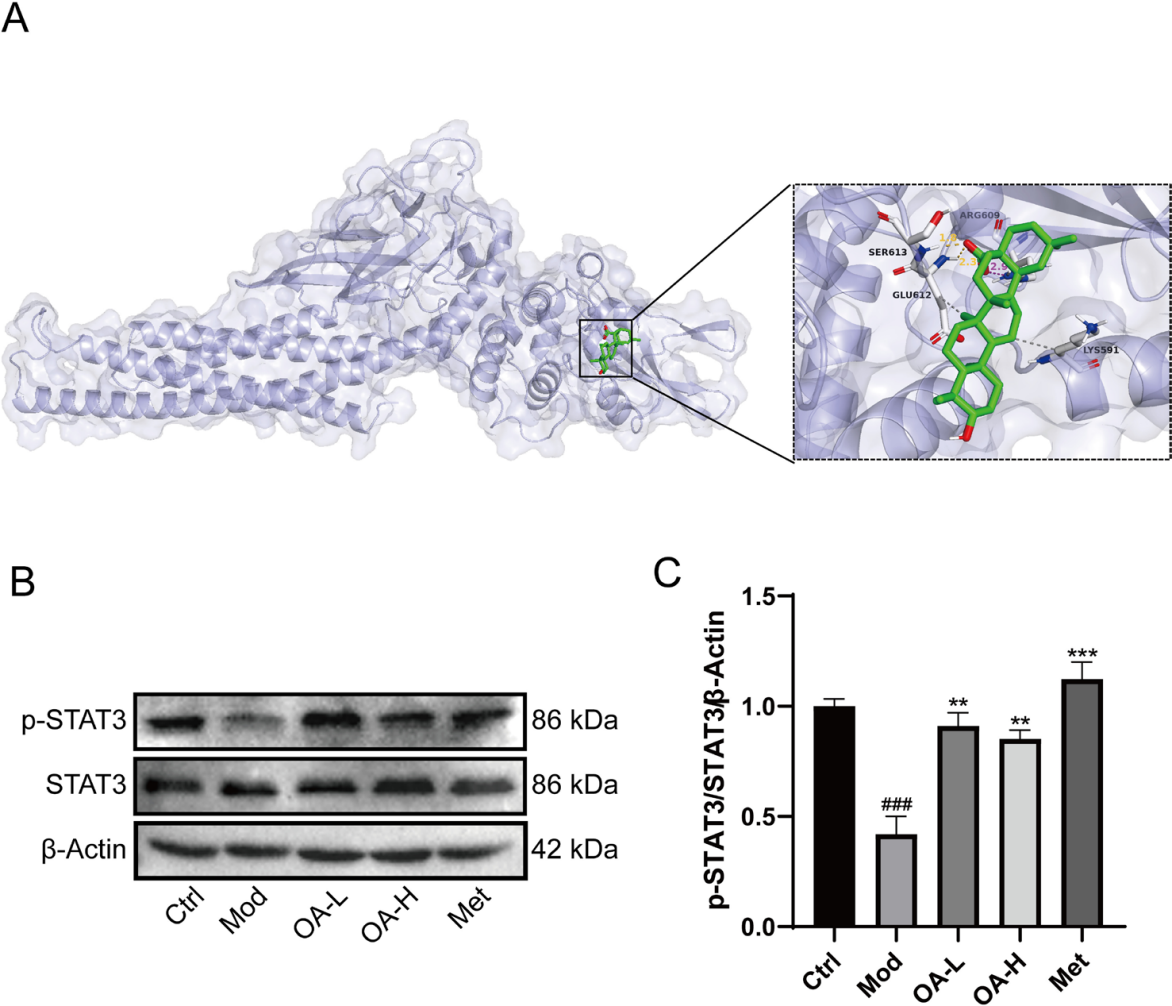
Improvement of T2DM by OA is associated with STAT3 phosphorylation. (A) The molecular docking analysis showing the interaction between OA and STAT3. (B) Western blot analysis assessed STAT3 protein expression and phosphorylation levels in liver samples. β‐Actin served as a loading control. (C) The quantitative data obtained from the western blot analysis, *n* = 3. Values were presented as mean ± SEM. ^###^ *p* < 0.001 versus the Ctrl group; **p < 0.01, ***p < 0.001 versus the Mod group.

Our earlier study identified the critical function of STAT3 in ensuring glycemic control in the context of T2DM, as well as the therapeutic potential of STAT3 in ginsenoside Rb1‐ameliorated T2DM (Wang, Liang, et al. 2023). Nevertheless, the influence of STAT3 on improving disrupted liver lipid balance and autophagy through OA in T2DM mice remains unexplored. Molecular docking was performed across the three identified binding pockets to investigate the potential binding mode between OA and STAT3. The docking protocol was validated by redocking of the co‐crystallized ligand SD36 (RMSD = 1.88 Å). Among the three pockets, OA showed a more favorable binding free energy of −6.2 kcal/mol in Pocket 1, with its docking pose nearly covering all key residues constituting the pY +0 site. Specifically, potential hydrogen bonds were predicted between OA and residues Glu612 (2.3 Å) and Ser613 (1.8 Å), consistent with the pharmacophore features of this pocket. Additionally, a salt bridge was predicted between OA and Arg609. Notably, Glu612 and Lys591 also contributed to hydrophobic contacts, further enhancing binding stability. The synergistic effects of these hydrogen bonds, salt bridges, and hydrophobic interactions may collectively anchor OA within the STAT3 SH2 domain binding pocket, potentially modulating STAT3 phosphorylation and subsequent dimerization (Figure 6A). Western blot analysis confirmed a substantial reduction in STAT3 phosphorylation at Y705 in the T2DM setting versus controls, but a considerable increase following OA or metformin treatment (Figure 6B,C). These findings indicate that the improvement of T2DM by OA is associated with STAT3 phosphorylation.

### 3.6 OA May Alter Autophagy‐Related Protein Expression in Insulin‐Resistant Cells in Association With STAT3 Phosphorylation

**FIGURE 7:**
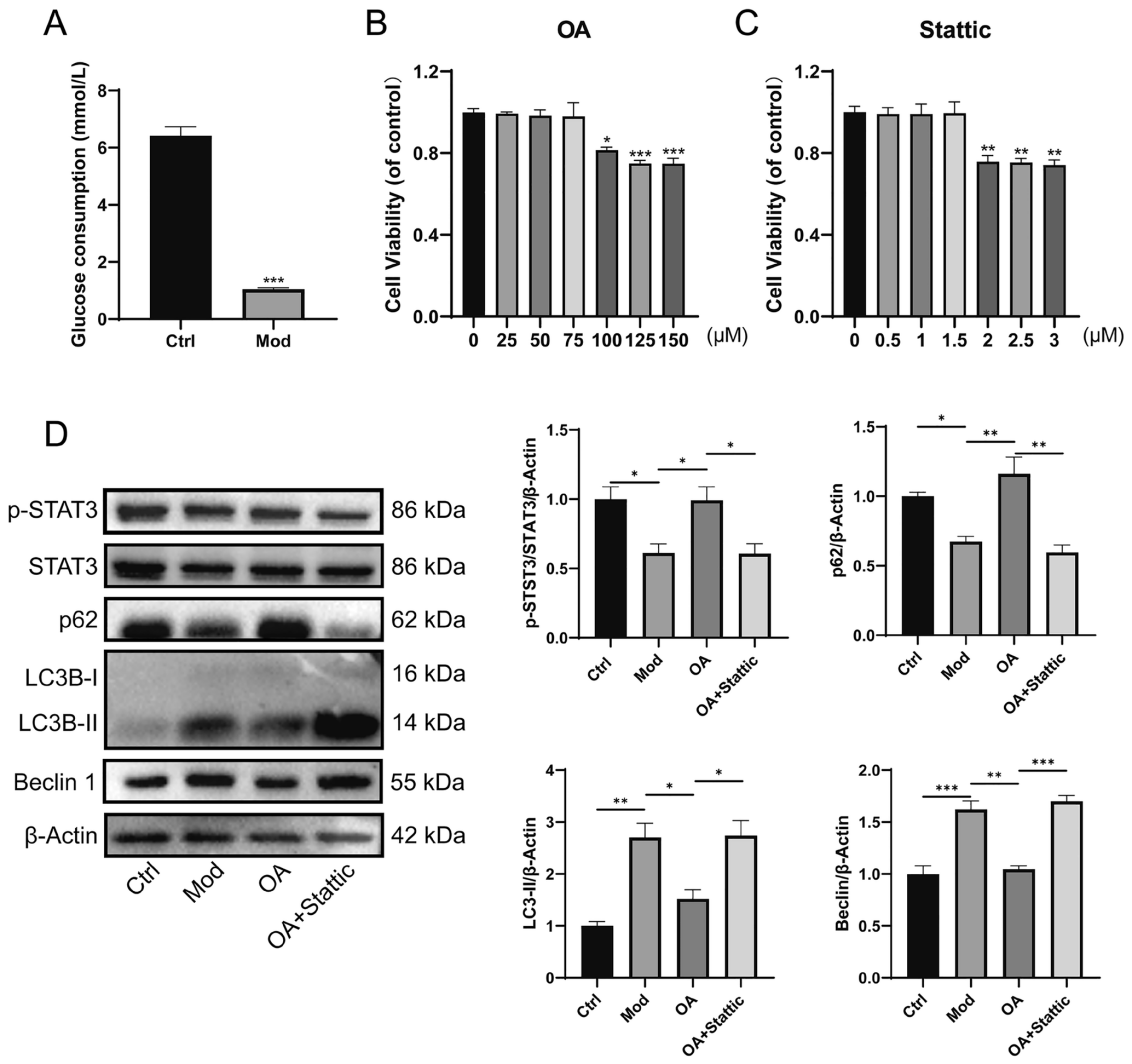
OA may alter autophagy‐related protein expression in insulin‐resistant cells in association with STAT3 phosphorylation. (A) Glucose consumption in control and model HepG2 cells. (B) Impact of varying OA concentrations on the HepG2 cell viability. (C) Impact of varying Stattic concentrations on the HepG2 cell viability. (D) The expressions of p‐STAT3, STAT3, p62, LC3, Beclin‐1, and β‐Actin were analyzed in HepG2 cells. β‐Actin served as a loading control. The bar graph presents the statistical data derived from the western blot analysis. *n* = 3. Data are expressed as mean ± SEM. *p < 0.05, **p < 0.01, ***p < 0.001.

To further explore whether OA ameliorates T2DM by STAT3‐regulated liver autophagy, we conducted in vitro experiments for further validation. To model insulin resistance, a hallmark of T2DM, glucosamine exposure was conducted on HepG2 cells (Wang, Liang, et al. 2023). Relative to the controls, the Mod group showed markedly reduced glucose consumption, validating the insulin‐resistant phenotype (Figure 7A). The effects of OA and Stattic, a commonly used STAT3 inhibitor, on HepG2 cell viability were determined using the CCK8 assay to ascertain their optimal concentrations (Figure 7B,C). Western blot assays were carried out to assess the expressions of STAT3 and autophagy‐linked proteins in the Ctrl, Mod, OA‐treated, and OA + Stattic‐treated groups. Additionally, OA treatment resulted in higher p62 levels and lower Beclin‐1 and LC3‐II expression in model cells; these changes were negated by Stattic co‐treatment (Figure 7D). Our study found that STAT3 phosphorylation at Y705 was also notably lower in the Mod group compared with controls. OA administration restored this phosphorylation, an effect counteracted by Stattic (Figure 7D). These findings suggest that OA may alter autophagy‐related protein expression in insulin‐resistant HepG2 cells in a manner associated with STAT3 phosphorylation.

### 3.7 OA May Regulate Fatty Acid Synthesis, Uptake, and β‐Oxidation in Insulin‐Resistant Cells in Association With STAT3 Phosphorylation

**FIGURE 8:**
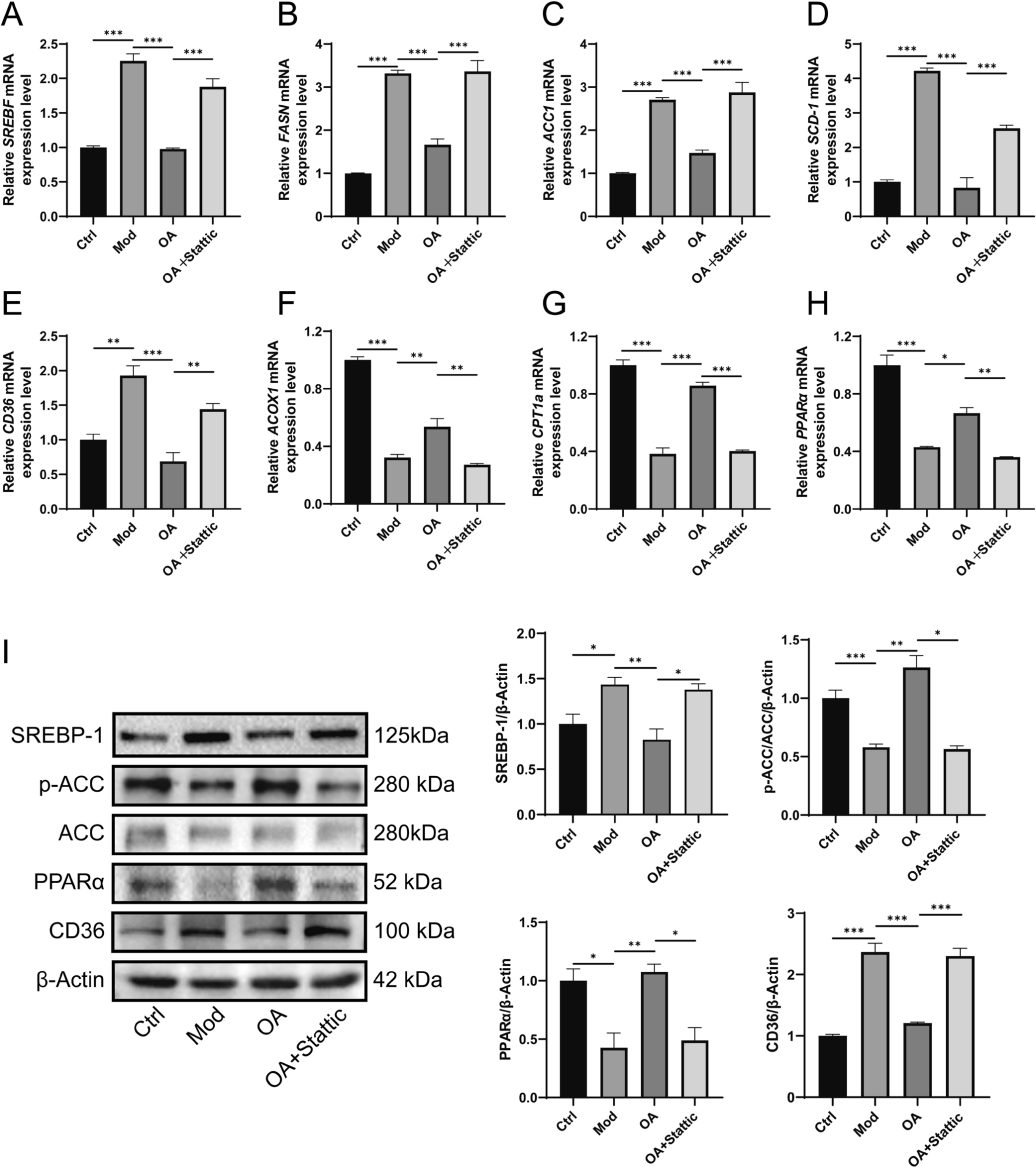
OA may regulate fatty acid synthesis, uptake, and β‐oxidation in HepG2 insulin‐resistant cells in association with STAT3 phosphorylation. (A–D) The levels of fatty acid synthesis‐related genes. (E) The gene expression of *CD36*. (F–H) The levels of fatty acid β‐oxidation‐related genes. (I) The protein expressions of SREBP‐1, p‐ACC, ACC, PPARα, CD36, and β‐Actin were analyzed in various HepG2 cell groups, with a bar graph presenting the quantitative results. β‐Actin served as a loading control. *n* = 3. Data are expressed as mean ± SEM. *p < 0.05, **p < 0.01, ***p < 0.001.

Subsequently, we pursued further analysis to explore whether OA modulates hepatic fatty acid metabolism in the insulin‐resistant liver cells in association with STAT3 phosphorylation, thereby improving T2DM. On one hand, the Mod group exhibited significantly elevated expression of genes linked to fatty acid uptake and synthesis, including *SREBF*, *FASN*, *ACC1*, *SCD‐1*, and *CD36* relative to the Ctrl group. These expression levels exhibited notable reduction following OA treatment, whereas co‐treatment with Stattic reversed this effect (Figure 8A–E). On the other hand, relative to controls, the Mod group displayed a pronounced decline in *ACOX‐1*, *CPT1a*, and *PPARα* mRNA expression, which are genes that are related to fatty acid β‐oxidation, and OA treatment notably reversed this effect (Figure 8F–H). Similarly, co‐treatment with OA and Stattic down‐regulated the expression of these genes (Figure 8F–H). Western blotting confirmed that there was a notable increase in the levels of SREBP‐1 and CD36 expression in the Mod group, and that ACC phosphorylation at Ser79 and PPARα protein levels were diminished, relative to the Ctrl group. Conversely, OA‐treated cells showed reduced SREBP‐1 and CD36 expression, with increased ACC phosphorylation and PPARα expression (Figure 8I). Co‐treatment with OA and Stattic elevated SREBP‐1 and CD36 protein expressions, while decreasing p‐ACC and PPARα levels (Figure 8I). These findings suggest that OA is likely to modulate lipid metabolism‐related gene and protein expression in insulin‐resistant HepG2 cells in a manner sensitive to STAT3 inhibition.

## 4 Discussion

T2DM can lead to severe complications, including cardiovascular diseases, retinopathy, and kidney diseases, posing a significant health challenge globally. Despite the availability of various treatments, the management of T2DM still faces certain challenges. Traditional anti‐diabetic medications, including biguanides, sulfonylureas, SGLT2 inhibitors, thiazolidinediones, and α‐glucosidase inhibitors, can lead to adverse effects such as hypoglycemia, hepatic damage, nausea, vomiting, weight gain, and urinary or genital tract infections (Chatterjee et al. 2017; Chitturi and George 2002). Therefore, advancements in T2DM pharmacotherapy call for safer and more effective agents. Our study investigates how OA works to treat T2DM, offering new perspectives for its use in clinical practice.

Traditional Chinese medicine provides effective alternatives for preventing and treating diabetes mellitus. *Ligustri Lucidi* Fructus, the dried ripe fruit of *Ligustrum lucidum* Ait. (Oleaceae), represents a traditional Chinese medicinal herb with established hypoglycemic efficacy. OA serves as its principal bioactive constituent (Cao et al. 2023). This natural pentacyclic triterpenoid has attracted significant research interest for its distinctive chemical structure and diverse medicinal actions. More importantly, within the experimental dosage range, OA does not exhibit significant hepatotoxicity (Wang et al. 2013). In diabetic nephropathy rats, OA can improve lipid accumulation and structural abnormalities in the kidneys of diabetic rats, effectively improving renal function and providing significant protection against chronic kidney disease (Liu et al. 2022). Additionally, the combination treatment of metformin and OA has been shown to improve glucose tolerance and insulin sensitivity by inhibiting gluconeogenesis and promoting glycogen synthesis, thus alleviating symptoms in *db/db* mice (Wang et al. 2015). Mechanistically, OA may ameliorate fructose‐induced hepatic fat deposition by suppressing SCD1 gene expression and enhancing fatty acid oxidation (Yao et al. 2023). OA can also reduce lipid accumulation in differentiated adipocytes through decreasing the expressions of PPAR‐γ and C/EBP‐α (Sung et al. 2010). Furthermore, OA shields the liver from ischemia–reperfusion damage by down‐regulating autophagy‐related proteins Beclin‐1 and LC3 (Wang et al. 2019). In keratinocytes, OA mitigates autophagy induced by aryl hydrocarbon receptor activation, protecting against skin aging initiated by particulate matter (Kim et al. 2021). Therefore, it is evident that OA can ameliorate T2DM by modulating multiple pathways, cellular processes, and tissues, warranting further investigation.

This study elucidates the mechanistic role of OA in regulating hepatic autophagy and steatosis in T2DM mice. The pathogenesis of hepatic lipid deposition encompasses increased DNL and fatty acid uptake, along with reduced fatty acid oxidation (Ipsen et al. 2018). DNL serves as the primary pathway that converts the key metabolic intermediate acetyl‐CoA into newly formed fatty acids, with increased DNL potentially causing hepatic steatosis, hypertriglyceridemia, and steatohepatitis. SREBP1c serves as a central regulator of fatty acid synthesis at the transcriptional level, which indirectly worsens hepatic insulin resistance by enhancing DNL and producing harmful lipids (Ipsen et al. 2018). Research indicates that increased expression of genes linked to DNL, including *SREBP‐1c*, *FASN*, *ACC1*, and *SCD‐1*, results in fatty acid accumulation in hepatocytes (Kohjima et al. 2007; Yao et al. 2023). Additionally, CD36 acts as a transporter for fatty acids, facilitating its uptake (Ipsen et al. 2018). Hepatic CD36 overexpression markedly enhances fatty acid uptake, contributing to greater lipid storage within the liver (Koonen et al. 2007). Consistently, in animals fed a HFD, hepatic CD36 knockout improves liver steatosis, insulin sensitivity, and systemic inflammation (Wilson et al. 2016). Besides, in T2DM mice, the expression of genes involved in fatty acid β‐oxidation, such as *ACOX‐1*, *PPAR*α, *and CPT1a*, is decreased. This down‐regulation results in liver lipid metabolism disorders (Wang et al. 2023). Consistent with these findings, reduced expression of CPT1a and PPARα also results in the accumulation of fatty acids in hepatocytes (Kohjima et al. 2007). Our findings demonstrated severe hepatic lipid metabolism disorder in T2DM mice. This pathological change is linked to the notable up‐regulation of lipogenesis and lipid uptake‐related genes and proteins, including SREBP‐1c, ACC1, FASN, SCD1, and CD36, alongside the significant down‐regulation of genes and proteins linked to fatty acid β‐oxidation, such as ACOX‐1, PPARα, and CPT1a. Notably, treatment with OA significantly reversed these alterations. Additionally, findings from insulin‐resistant HepG2 cells aligned with those seen in mice. Therefore, our study suggests that OA treatment might alleviate liver lipid metabolism disorder and ameliorate T2DM by lowering DNL and lipid uptake and promoting fatty acid β‐oxidation.

Autophagy balances cellular processes through the disposal and recycling of cytoplasmic contents and proteins that are misfolded. Maintaining autophagy homeostasis can prevent various pro‐diabetic factors, including oxidative stress, endoplasmic reticulum stress, lipotoxicity, and glucotoxicity. Moreover, autophagy is closely associated with β‐cell damage and insulin resistance, which are central characteristics of T2DM (Sehrawat et al. 2023). Autophagy is precisely controlled by a group of proteins. LC3 exists in two forms: LC3‐I and LC3‐II, which serve as distinctive molecular markers for autophagosomes. Autophagosome formation involves the cleavage of LC3‐I by the E1‐like enzyme Atg7, followed by its transfer to the E2‐like enzyme Atg3, and subsequent conjugation with phosphatidylethanolamine to produce LC3‐II. This conversion is usually considered a sign of autophagy occurrence. Therefore, the elevated LC3‐II expression typically signifies enhanced autophagosome formation and serves as a marker for autophagy occurrence (Li et al. 2021). P62 functions as a cargo receptor in autophagy, identifying and directing target proteins or organelles to autophagosomes for degradation (Komatsu 2022). Damage to autophagy is usually accompanied by the accumulation of p62 (Mizushima and Komatsu 2011). Beclin‐1 is involved in autophagy initiation. It forms a complex by binding to PIK3C3/VPS34 and Atg14, promoting the formation, extension, and maturation of autophagosomes. Decreased Beclin‐1 expression diminishes autophagy activity. Conversely, overexpression of Beclin‐1 protein will increase autophagy flux (Li et al. 2021; Yamamoto et al. 2018). Autophagy is integral to numerous physiological and pathological processes and is crucial in the treatment of various diseases. However, its effects are double‐edged, which may be related to diseases and tissue types. This complexity brings challenges to research. Especially in the research field of T2DM, there are still some contradictory results in the current literature. For example, in muscle and adipose tissues, autophagy activation enhances insulin function; however, in pancreatic β‐cells, prolonged activation of autophagy can result in reduced insulin release due to the excessive degradation of insulin granules (Yamamoto et al. 2018). Similarly, autophagy exhibits a dual role in the liver. Under physiological conditions, autophagy maintains hepatic metabolic homeostasis by promoting lipid droplet breakdown through lipophagy and eliminating damaged mitochondria via mitophagy. When acute liver injury occurs, autophagy is markedly activated and exerts a protective effect by removing damaged organelles, attenuating oxidative stress, and suppressing inflammatory responses (Ke 2019). However, in NAFLD patients and corresponding mouse models, the presence of hepatic steatosis disrupts autophagic flux, a disturbance that correlates closely with heightened endoplasmic reticulum stress and apoptosis (González‐Rodríguez et al. 2014). On the other hand, at the cirrhosis stage, elevated LC3B levels, enhanced colocalization of LC3B with LAMP1, and upregulated expression of LAMP2 and cathepsin D are observed in patient liver samples, indicating hyperactivation of the autophagy‐lysosomal system. Notably, autophagy inhibition by chloroquine attenuates CCl_4_‐induced ductular reaction and fibrosis (Hung et al. 2015), suggesting a profibrogenic role of autophagy under these conditions. Furthermore, autophagy has been reported to promote the formation of hepatocyte growth factor‐directed Axin2^+^CD90^+^ cancer stem cells, thereby driving hepatocarcinogenesis in the cirrhotic microenvironment (Li et al. 2017). Additionally, a study indicates that OA administration dose‐dependently decreases autophagy marker protein expression in mouse kidneys, potentially due to its antioxidant properties (Potočnjak et al. 2019). This suggests that variations in cell types, pathological contexts, and stages of disease progression may collectively contribute to the biphasic effect of autophagy in T2DM. In light of these divergent observations, our experiment revealed a significant increase in LC3‐II and Beclin‐1 protein expression, with a simultaneous decrease in p62, in the livers of T2DM mice and insulin‐resistant HepG2 cells. However, these findings alone are insufficient to determine whether such alterations in autophagy‐associated proteins represent excessive activation or a compensatory protective response within this specific T2DM model. Notably, OA treatment restored the expression of these proteins toward control levels, suggesting a therapeutic effect through the re‐establishment of autophagic homeostasis. To our knowledge, our study is the first to demonstrate that OA may ameliorate T2DM by modulating autophagy‐related pathways, thereby revealing a potential target for further investigation.

STAT3 has been increasingly proven to play important roles in various metabolic diseases (Inoue et al. 2004; Wang, Liang, et al. 2023). Few findings have indicated a link between STAT3 and both lipid metabolism and autophagy, though the underlying mechanism remains unclear. IL‐17 administration in keloid fibroblasts elevates expressions of p‐STAT3 (Y705), p62, and LC3II/I, resulting in autophagosome accumulation but significantly impairs autolysosome formation, indicating a defect in autophagy function (Lee et al. 2022). PM2.5 intensifies inflammation and boosts lipid buildup in macrophages via suppression of the JAK2/STAT3 axis (Yang et al. 2021). Carbenoxolone enhances the hepatic level of p‐STAT3 by activating the JAK2/STAT3 signaling, which subsequently decreases SREBP‐1c and FAS expressions, thereby protecting the liver against HFD‐induced lipid metabolism disorders (Chen et al. 2019). It is noteworthy that STAT3 signaling exhibits significant context‐dependent pleiotropy. In obesity‐associated adipose tissue inflammation, STAT3 activation in T cells promotes IFN‐γ production and macrophage infiltration, exacerbating insulin resistance (Priceman et al. 2013); whereas in diquat‐induced acute liver injury, hepatocyte STAT3 activation exerts protective effects through antioxidant defense and anti‐apoptotic mechanisms mediated by HO‐1 upregulation and Bax/Bcl‐2 balance restoration (Li et al. 2025). Furthermore, persistent STAT3 activation during hepatic fibrosis and hepatocellular carcinoma progression promotes hepatic stellate cell activation, tumor proliferation, and sorafenib resistance (Jiang et al. 2021; Sun et al. 2024). The present study observed an association between OA treatment and STAT3 phosphorylation coinciding with restored autophagic markers and metabolic parameters, suggesting a protective role of STAT3 activation in this experimental context. Nonetheless, the dynamic effects of prolonged OA intervention on STAT3 signaling and its potential divergent roles across different disease stages and tissue types warrant further investigation. The present study observed an association between OA treatment and STAT3 phosphorylation coinciding with restored autophagic markers and metabolic parameters, suggesting a protective role of STAT3 activation in this experimental context. However, the long‐term impact of OA treatment on STAT3 signaling, as well as its possibly varying functions in different stages of disease and types of tissues, requires additional research. This study is the first to suggest that OA may regulate hepatic lipid metabolism and autophagy in association with STAT3 phosphorylation in T2DM. Molecular docking, validated by redocking of the co‐crystallized ligand SD36 (RMSD = 1.8 Å), revealed a favorable binding affinity between OA and STAT3 (binding energy: −6.2 kcal/mol in the pY +0 pocket). This suggests that OA may bind to the STAT3 SH2 domain, which warrants further experimental investigation. Western blot analysis confirmed that OA activates STAT3. In vitro experiments demonstrated that OA inhibited fatty acid uptake and lipogenesis, enhanced fatty acid β‐oxidation, and altered autophagy‐related protein expression in insulin‐resistant HepG2 cells‐effects that were reversed by the STAT3 inhibitor Stattic. These findings support a role for STAT3 in maintaining lipid metabolism and autophagy homeostasis, suggesting its involvement in OA's therapeutic effects in T2DM.

This study has some limitations that should be recognized. First, regarding sex as a biological variable: only male C57BL/6J mice were used, and the results may not generalize to females due to well‐documented sex differences in metabolic responses, including HFD‐induced steatosis, insulin resistance, STZ sensitivity, and autophagy regulation. Female mice are generally protected from diet‐induced metabolic dysfunction due to estrogen‐mediated effects on lipid metabolism and insulin sensitivity, and the estrous cycle can further modulate autophagic activity and inflammatory responses (García‐Macia et al. 2019; B. Kim et al. 2020; Stubbins et al. 2012). Consequently, it is still unclear whether OA provides similar protective benefits in female T2DM mice, and if these effects differ depending on the stage of the estrous cycle, which requires further study. Second, the HFD + low‐dose STZ model combines diet‐induced insulin resistance with chemical β‐cell injury and may not fully recapitulate the pathophysiology of pure T2DM. Consequently, some of the observed protective effects of OA‐especially those related to STAT3 activation and autophagy modulation‐might, at least in part, reflect cytoprotection against STZ‐induced cellular stress rather than solely amelioration of insulin resistance per se. Third, in terms of evaluating autophagy: our interpretation relied on static protein levels (p62, LC3‐II, Beclin‐1); assessment of autophagic flux (using lysosomal inhibitors or mRFP‐GFP‐LC3 reporters) and upstream/lysosomal markers (e.g., ULK1, LAMP1/2) would provide more definitive mechanistic insights. Fourth, regarding the STAT3 mechanism: while OA increased STAT3 phosphorylation and the inhibitor Stattic reversed OA's effects in vitro, we lack in vivo genetic evidence (e.g., liver‐specific STAT3 knockout) and direct binding assays (SPR/ITC/CETSA) to confirm direct OA‐STAT3 interaction. Due to Stattic's known off‐target effects and the absence of evaluation on upstream kinase (JAK2) or cytokine receptors, it is not possible to definitively determine whether OA acts directly on STAT3 or through indirect pathways. Fifth, regarding operational limitations: sample sizes varied across assays based on technical quality control and practical constraints, as detailed in Section 2.10. Sixth, regarding in vitro models: HepG2 cells are transformed and may not fully represent normal hepatocyte metabolism, and glucosamine‐induced insulin resistance captures only one aspect of the complex pathophysiology. Validation in primary hepatocytes or alternative insulin resistance models (e.g., palmitate/oleate‐induced lipotoxicity) would strengthen the generalizability of our findings. Finally, although we monitored gross pancreatic status by evaluating pancreas weight, fasting insulin levels, insulin during OGTT, and HOMA‐IR‐key parameters for assessing insulin resistance and β‐cell function‐were not measured due to sample availability constraints. While our IPITT results provide functional evidence of improved systemic insulin sensitivity following OA treatment, the absence of these specific insulin‐related measurements limits a more comprehensive mechanistic interpretation. Future studies should include these assessments to further validate our findings.

## 5 Conclusion

Collectively, these data indicate that OA ameliorates aberrant hepatic lipid metabolism and autophagy in the context of T2DM, with concomitant STAT3 phosphorylation. Both low‐dose (20 mg/kg/day) and high‐dose (60 mg/kg/day) OA treatments exerted comparable therapeutic effects on glycemic control, hepatic steatosis, and autophagy regulation, indicating that the lower dose may be sufficient to achieve near‐maximal efficacy under the current experimental conditions. This lack of clear dose dependency may reflect a pharmacological plateau, a phenomenon commonly observed with natural products that have pleiotropic mechanisms. These findings provide a theoretical foundation for developing novel strategies in treating T2DM. A comprehensive strategy is urgently needed to prevent and treat T2DM, emphasizing the control of hyperglycemia and obesity, along with preventing diabetes‐related complications. Based on the current research, it is reasonable to speculate that combining OA with other T2DM medications may prove to be a superior approach in combating T2DM. Therefore, further exploration of the detailed mechanisms of OA in T2DM treatment and its potential clinical applications is highly important.

## Author Contributions

**Zirui Ji:** investigation, data curation, formal analysis, writing – original draft. **Yuanfeng Huang:** investigation, data curation, formal analysis. **Xiangjin Pu:** investigation. **Jinqing Feng:** investigation. **Yiwen Liao:** investigation. **Wanfen Lin:** investigation. **Qing Zhu:** writing – review and editing. **Weixuan Wang:** conceptualization, funding acquisition, writing – review and editing.

## Funding

This work was supported by National Natural Science Foundation of China [grant number 82300927], China Association for Science and Technology Youth Talent Lift Project [grant number 2024‐2026QNRC001], and Research team project of prevention and treatment of diabetic cardiomyopathy with integrated Chinese and Western medicine [grant number 2024ZZ06].

## Ethics Statement

The animal testing was conducted in strict accordance with the National Research Council's Guide for the Care and Use of Laboratory Animals and received formal approval from the Animal Ethics Committee of Guangdong Pharmaceutical University (Approval Number gdpulacspf2022195).

## Conflicts of Interest

The authors declare no conflicts of interest.

## Supporting information


**Figure S1:** Effect of OA on pancreas weight in T2DM.

**Table S1:** The Sequences of qPCR Primers.

## Data Availability

Data will be made available on request.
