# Supplementary material for: Oleanolic Acid Ameliorates Hepatic Lipid Metabolism and Autophagy in Type 2 Diabetic Mice via the STAT3 Signaling Pathway
Source: Food Sci Nutr. 2026 Jul 15;14(7):e72063. doi: 10.1002/fsn3.72063 (PMC13373316; doi:10.1002/fsn3.72063)
Supplement: Supplementary file 1 — Figure S1: Effect of OA on pancreas weight in T2DM. [file FSN3-14-e72063-s001.docx]

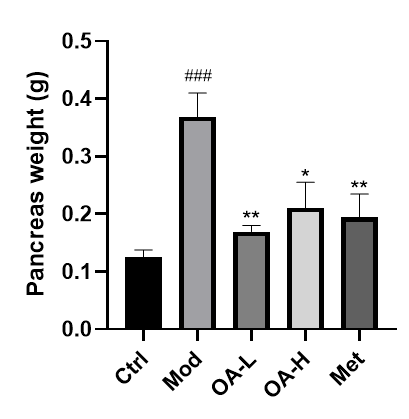


**Supplementary Figure 1. Effect of OA on pancreas weight in T2DM.**

Data are presented as mean ± SEM (n = 6 per group). Ctrl: normal control group; Mod: T2DM model group; OA-L: T2DM + low-dose OA (20 mg/kg) ; OA-H: T2DM + high-dose OA (60 mg/kg) ; Met: T2DM + metformin (250 mg/kg). *^###^P* < 0.001 versus Ctrl;*^**^P* < 0.01 versus Mod. *^*^P* < 0.05 versus Mod.
