# Supplementary material for: Oleanolic Acid Ameliorates Hepatic Lipid Metabolism and Autophagy in Type 2 Diabetic Mice via the STAT3 Signaling Pathway
Source: Food Sci Nutr. 2026 Jul 15;14(7):e72063. doi: 10.1002/fsn3.72063 (PMC13373316; doi:10.1002/fsn3.72063)
Supplement: Supplementary file 2 — Table S1: The Sequences of qPCR Primers. [file FSN3-14-e72063-s002.docx]

**Table S1. The Sequences of qPCR Primers**

| Species | Gene | Sequences |
| --- | --- | --- |
| Human | *FASN* | F 5’-CCGAGACACTCGTGGGCTA-3’ |
|  |  | R 5’-CTTCAGCAGGACATTGATGCC-3’ |
| Human | *ACC1* | F 5’-ATGTCTGGCTTGCACCTAGTA-3’ |
|  |  | R 5’-CCCCAAAGCGAGTAACAAATTCT-3’ |
| Human | *SCD-1* | F 5’-GCCCCTCTACTTGGAAGACGA-3’ |
|  |  | R 5’-AAGTGATCCCATACAGGGCTC-3’ |
| Human | *SREBF* | F 5’-GCCCCTGTAACGACCACTG-3’ |
|  |  | R 5’-CAGCGAGTCTGCCTTGATG-3’ |
| Human | *ACOX-1* | F 5’-ACTCGCAGCCAGCGTTATG-3’ |
|  |  | R 5’-AGGGTCAGCGATGCCAAAC-3’ |
| Human | *PPARα* | F 5’-CTGCCTGCTCCACCTTT-3’ |
|  |  | R 5’-CATAGCTCCTCTCCCCTCA-3’ |
| Human | *CPT1a* | F 5’-TCCAGTTGGCTTATCGTGGTG-3’ |
|  |  | R 5’-TCCAGAGTCCGATTGATTTTTGC-3’ |
| Human | *CD36* | F 5’-GGCTGTGTTTGGAGGTATTC-3’ |
|  |  | R 5’-TTCTGTGCCTGTTTTAACCC-3’ |
| Human | *ACTB* | F 5’-CATGTACGTTGCTATCCAGGC-3’ |
|  |  | R 5’-CTCCTTAATGTCACGCACGAT-3’ |
| Mouse | *Fasn* | F 5’-AAGCGGTCTGGAAAGCTGAA-3’ |
|  |  | R 5’-AGGCTGGGTTGATACCTCCA-3’ |
| Mouse | *Acc1* | F 5’-GCCTTTCACATGAGATCCAGC-3’ |
|  |  | R 5’-CTGCAATACCATTGTTGGCGA-3’ |
| Mouse | *Scd-1* | F 5’-CCTGCCTCTTCGGGATTTT-3’ |
|  |  | R 5’-GCCCATTCGTACACGTCATT-3’ |
| Mouse | *Srebf* | F 5’-GGAGCCATGGATTGCACATT-3’ |
|  |  | R 5’-GGCCCGGGAAGTCACTGT-3’ |
| Mouse | *Cd36* | F 5’-TTAATGGCACAGACGCAGCC-3’ |
|  |  | R 5’-GGATTCTGGAGGGGTGATGC-3’ |
| Mouse | *Acox-1* | F 5’-CGGAAGATACATAAAGGAGACC-3’ |
|  |  | R 5’-AAGTAGGACACCATACCACCC-3’ |
| Mouse | *Pparα* | F 5’-CATTTCTCCTTGGCGTGT-3’ |
|  |  | R 5’-CCTCAGACCTTGCTTTGG-3’ |
| Mouse | *Cpt1a* | F 5’-AGGACCCTGAGGCATCTATT-3’ |
|  |  | R 5’-ATGACCTCCTGGCATTCTCC-3’ |
| Mouse | *Actb* | F 5’-GCTCCGGCATGTGCAAAG-3’ |
|  |  | R 5’-TTCCCACCATCACACCCTGG-3’ |
